# Association between passive tobacco exposure and caries in children and adolescents. A systematic review and meta-analysis

**DOI:** 10.1371/journal.pone.0202497

**Published:** 2018-08-16

**Authors:** Lourdes González-Valero, José María Montiel-Company, Carlos Bellot-Arcís, Teresa Almerich-Torres, José Enrique Iranzo-Cortés, José Manuel Almerich-Silla

**Affiliations:** Stomatology Department, University of Valencia, Spain; Universidade Federal de Sao Paulo, BRAZIL

## Abstract

To examine the available evidence on the association between exposure to tobacco use in the womb and in infancy and the presence of caries in primary and permanent dentition in children and adolescents.

A systematic review was conducted through searches in 4 data bases (Pubmed, Scopus, Embase and Web of Science), complemented by hand-searching. Of the 559 articles identified, 400 were duplicates. Finally, 28 articles were included in the qualitative review and 21 in the meta-analysis. Their quality was assessed using the Newcastle-Ottawa scale. The quality was medium in 44% of the articles included and high in 56%.

The overall meta-analysis gave a significant odds ratio (OR = 1.53, 95% confidence interval 1.39–1.68, Z test p-value = 0.000) and high heterogeneity (*Q* = 200.3, p = 0.000; I^2^ = 86.52%). Separate meta-analyses were also performed for three subgroups: exposure in the womb (prenatal) and caries in primary dentition, which resulted in a significant OR = 1.46 with a 95% CI of 1.41–1.52 (Z test p = 0.000), without heterogeneity (*Q* = 0.91, p = 0.824; I^2^ = 0%); exposure in infancy (postnatal) and caries in primary dentition, with OR = 1.72 (95% CI 1.45–2.05) and high heterogeneity (*Q* = 76.59, p = 0.00; I^2^ = 83.01%); and postnatal exposure and caries in permanent dentition, with OR = 1.30 (95% CI 1.25–1.34) and no heterogeneity (*Q* = 4.48, p = 0.880; I^2^ = 0%). In children and adolescents, a significant though moderate association was found between passive tobacco exposure and caries.

## Introduction

Dental caries is still one of the main diseases of the oral cavity, affecting a third of the population in the world[1]. Caries prevalence has fallen in industrialised countries in recent years, although it has increased in developing countries. The current epidemiological pattern of dental caries in the child population of developed countries shows lower caries levels but an unbalanced distribution, concentrated in the most socially-disadvantaged sector of the population[2]. The economic and social cost of caries is very high. Dental caries is considered a pandemic due to its global distribution and severe consequences[3].

Dental caries is a biofilm-mediated, sugar-driven, multifactorial, dynamic disease that results in the phasic demineralization and remineralization of dental hard tissues. The balance between pathological and protective factors influences the initiation and progression of caries[4]. The pathological factors include acidogenic bacteria, inhibition of salivary function, and frequency of ingestion of fermentable carbohydrates and the protective factors include salivary flow, numerous salivary components, antibacterials (both natural and applied), fluoride from extrinsic sources, and selected dietary components[5].

Over 1 billion people in the world smoke tobacco, and an enormous increase has been predicted, to 1.9 billion in 2025, with no great differences between genders. According to the WHO, smoking causes approximately 71% of lung cancer cases, 42% of chronic respiratory diseases and around 10% of cardiovascular diseases. Oral, throat and oesophagal cancers appear up to ten times more frequently in smokers than in non-smokers[3].

There is strong evidence that tobacco use has numerous negative effects on oral health, such as staining of teeth and tooth restorations by nicotine and tar, reduced senses of smell and taste, oral diseases such as smoker’s palate and smoker’s melanosis, precancerous lesions such as leukoplakias, oral cancer, oral candidiasis due to reduced saliva flow, changes in the bacterial flora and periodontal disease[6]. The WHO estimates that nearly 700 million children are exposed to tobacco smoke, mostly from their parents in their own homes[7]. Tobacco smoke has not passed through a filter, so it contains far higher concentrations of harmful substances than smoke which is inhaled through a cigarette, and is therefore potentially more dangerous for non-smokers[6,8]

Exposure to tobacco smoke is a major cause of paediatric morbidity and mortality around the world[9]. Children exposed to large quantities of environmental smoke are more likely to suffer respiratory infections, ear blockages, asthma attacks, hospitalisation and irritation of the eyes, throat and respiratory tract. Tobacco smoke has also been related to dental caries in children and adolescents[7].

Some controversy surrounds the possible association between tobacco and caries in children. Hanioka et al.[10] stated that the overall evidence in epidemiological studies is that a causal association in early childhood caries is possible, but the evidence for permanent teeth and the effect of maternal smoking during pregnancy are insufficient. A systematic review by Slayton[11] concluded that there is limited evidence concerning the possible aetiology.

Consequently, the main objective was to examine the available scientific evidence concerning on the association between exposure to tobacco use in the womb and in infancy and the presence of caries in primary and permanent dentition in children and adolescents.

A systematic review and meta-analysis has been designed to answer the following PECO question: “Is Passive smoking (Exposure) associated with the development of cavitated lesions of caries in teeth (Outcome) of children and adolescents (Participants) compared with those not exposed (Comparison/control)?”.

## Materials and methods

### Study registration

The present study was registered in the International prospective register of systematic reviews (PROSPERO) with the registration id. CRD42028090177.

## Study selection criteria

The selection criteria for inclusion were: studies in humans in which the sample ages were children and adolescents (from 0 to 19 years-old), published since the year 2000, concerning passive tobacco exposure and dental caries. Unpublished resources were not considered. Longitudinal studies, case-control studies and cross-sectional studies were included provided they also had a control group of subjects not exposed to tobacco use for comparison and that the primary outcome of caries was expressed as odds ratios (or data provided on the number of events and totals for each group enabled the odds ratio to be calculated), or as hazard ratios or indices (DMFT, DMFS, dft or dfs).

## Search strategies and article selection

To identify the most relevant studies, irrespective of language, a thorough electronic search from January 2000 to 2^nd^ March 2018 was made in four databases: *Pubmed*, *Scopus*, *Embase and Web of Science*.

The search equation for Pubmed and Web of Science was: ((dental caries OR caries) AND (children OR adolescents) AND (Tobacco OR passive smoke OR passive smoking OR secondhand smoke OR secondhand smoking OR household smoke OR household smoking OR involuntary smoke OR involuntary smoking OR parental smoke OR parental smoking OR maternal smoke OR maternal smoking)). For Scopus, the equation was (TITLE-ABS-KEY (dental AND caries OR caries) AND TITLE-ABS-KEY (child OR adolescents) AND TITLE-ABS-KEY (passive AND smoking OR tobacco OR household AND smoking OR maternal AND smoking)) AND PUBYEAR > 1999 and finally, for Embase the search equation was 'dental caries' AND child AND smoking AND [2000–2018]/py.

Two calibrated reviewers (LGV and JMM-C) independently selected the articles. In the event of disagreement, they had to reach a consensus on whether or not to include the study in the review. Cohen’s kappa was used to determine the inter-reviewer reliability. The initial screening was performed by reading the titles and abstracts. If the information was insufficient, the decision was based on reading the full text of the article.

### Data mining

For each of the articles selected, the following information was recorded: authors, year of publication, type of study, sample size, sample age, prenatal tobacco exposure (tobacco smoking during the pregnancy) or postnatal tobacco exposure (unwanted breathing in of parents and relatives’ cigarette smoke by children who do not smoke at home), primary or permanent dentition, dental caries (defined as a tooth with a definite cavity and undermined enamel) as the primary outcome of interest expressed as odds ratios, hazard ratios or DMFT (number of decayed, missing or filled teeth in permanent dentition), DMFS (number of decayed, missing or filled surfaces in permanent dentition), dft (number of decayed or filled teeth in primary dentition) or dfs (number of decayed or filled surfaces in primary dentition) caries indices, and the existence or otherwise of a significant association.

### Study quality

The quality of the studies was assessed on the *Newcastle-Ottawa Scale*. This consists of three sections, each with a number of stars that can be awarded to the study. The three sections are *selection*, *comparability* and *outcome* or, for case-control studies, *exposure*. In the *selection* section, a cross-sectional study can obtain up to 5 stars but the maximum for cohort and case-control studies is 4 stars. All three types of study can achieve a maximum of 2 stars in the second section (*comparability*) and a maximum of 3 stars in the third section (*outcome/exposure*). The quality score is the sum of all the stars obtained by that study [12]. Articles with 8 or 9 stars were considered of high quality and those with 6 or 7 of medium quality; below 7 was considered poor quality.

### Quantitative analysis of the studies (meta-analysis)

The studies included in each meta-analysis were combined using the *random effects model*, assuming the inter-studies heterogeneity. The significance of the meta-analysis was assessed through the Z test. The heterogeneity was measured by the *I*^*2*^ *heterogeneity score*, the *Q statistic* and the *p-value*. A Q statistic with p<0.1 was considered to indicate heterogeneity. In addition, I^2^ classifies the heterogeneity as mild (I^2^ 25%-49%), moderate (50%-74%) or high (>75%).

For the presence of caries, the measure of effect size was the *odds ratio*, with 95% CI. When the study did not give the odds ratio, this was calculated from the events and totals. Whenever possible, the adjusted odds ratio was chosen. The estimate was considered significant when the Z test p-value was <0.05.

For the control of the possible heterogeneity sources, 3 meta-analysis divided into subgroups will be done, taking into account the dentition (primary or permanent) and the exposure (pre or postnatal).

The sensitivity of the meta-analysis was assessed by the one-study removed method, that assess the stability of the effect size obtained every time that a study is removed.

The publication bias in the meta-analysis was assessed through the *classic fail-safe number*, which estimates the number of statistically significant studies that would be required for a meta-analysis with a significant result (p<0.05) to cease to be significant, and through Egger’s regression intercept, when p<0.1. Additionally, symmetrical distribution of these studies was assessed through *funnel plots with Duval and Tweedie‘s Trim and Fill* that, after adjusting for missing studies, estimates a new overall effect size, that can be compared with the results obtained in the meta-analysis[13].

The meta-analyses were performed with *Comprehensive Meta-analysis v3*.*0* software (Biostat).

## Results

### Flow chart

On applying the search criteria, 149 articles were identified in PubMed, 44 in Scopus, 132 in Embase and 230 in Web of Science, totalling 555 articles; this was complemented by hand-searching.

**Fig 1:**
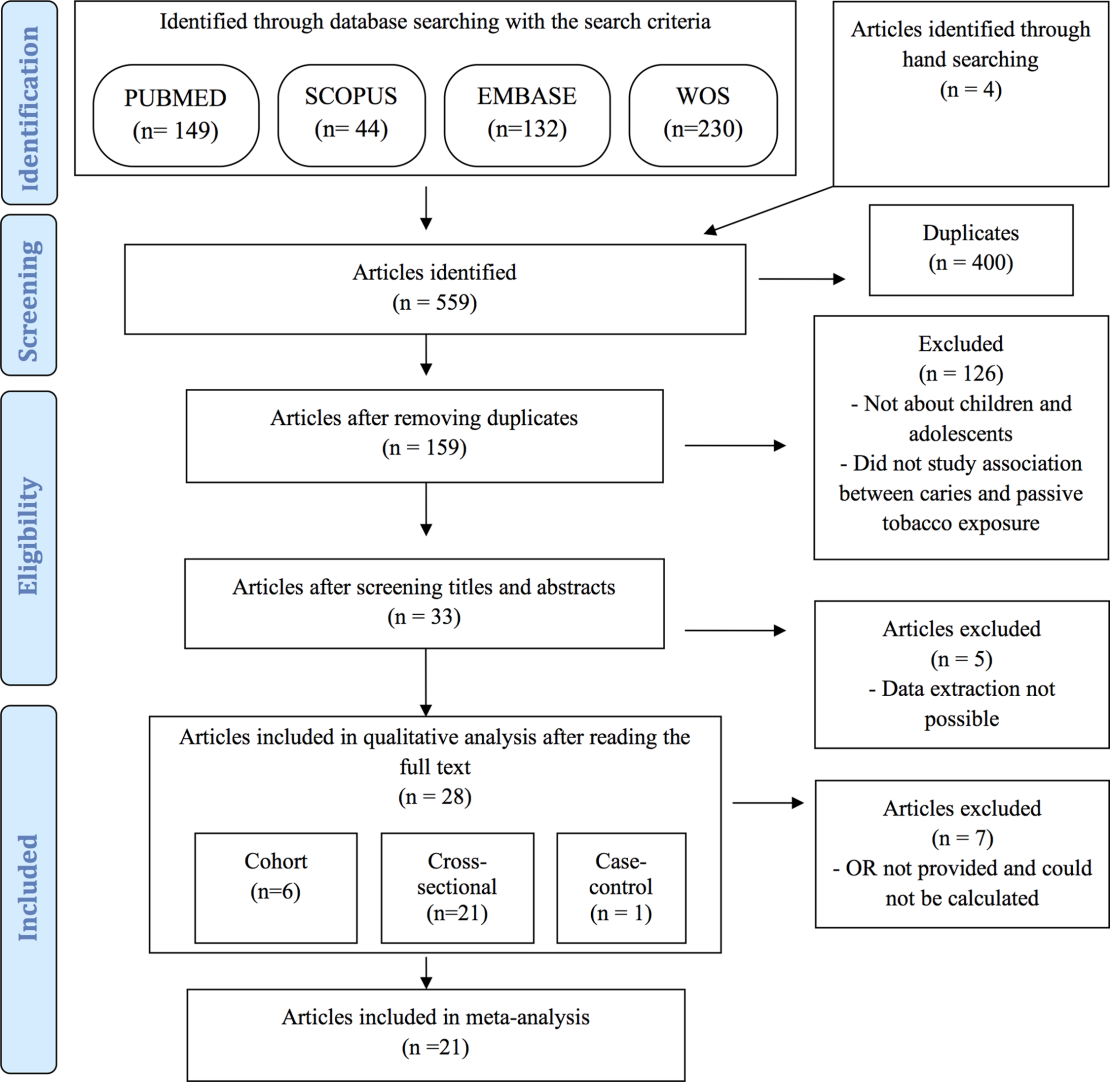
Flow diagram.

Following removal of 400 duplicate articles, 159 remained. Finally, 28 articles were included in the systematic review and 21 in the meta-analysis (Fig 1).

Cohen’s kappa value for the inter-reviewer reliability was 0.87

### Qualitative analysis

Of the articles selected for systematic review (Table 1), 21 were cross-sectional studies[14–34], one was a case-control study[35] and six were longitudinal studies[36–41].

**Table 1:** Characteristics of studies included in the review, measuring the association between passive exposure to tobacco use and the presence of caries in children and adolescents.

Study	Type	n	Age(yr)	Exposure	Dentition	Outcome	Association
Aida et al., 2008[14]	CS	3086	3	Postnatal	Primary	OR = 2.14 (1.59–2.87)	Yes
Aligne et al., 2003[15]	CS	3531	4 to 11	Postnatal	Primary	a: OR = 1.8 (1.2–2.7); ds	Yes
					Permanent	b: OR = 1.2 (0.8–1.85); DS	No
Almerich et al., 2013[16]	CS	889	12	Postnatal	Permanent	a: OR = 1.2 (0.82–1.75)	No
			15		Permanent	b: OR = 1.24 (0.84–1.83)	
Avçar et al., 2008[35]	CC	90	5	Postnatal	Primary	Cases: dft = 10.58±2.12	Yes
		90				Controls: dft = 4.64±2.91	
Ayo-Yusuf et al., 2007[17]	CS	1873	13 to 15	Postnatal	Permanent	Caries in 2^nd^ molars	Yes
						OR = 2.02 (1.22–3.33)	
Bakhurji et al., 2017 [33]	CS	294	12–15	Postnatal	Permanent	OR = 1.37 (0.46–4.08)	No
Bernabé et al., 2017[36]	L	1102	1–4	Postnatal	Primary	Data 4 years; Exposed dmfs = 5.18±9	Yes
						Non-exposed dmfs = 2.78 ±6.51	
Carbajosa et al., 2011[18]	CS	380	10 to 15	Postnatal	Permanent	Exposed DMFT = 1.62±2.2; df = 0.27±0.78	Yes
						Non-exposed DMFT = 0.92±1.40; df = 0.10±0.47	
Claudia et al., 2016 [41]	L	1687	0.5–6	Prenatal	Primary	RR = 1.42 (1.20–1.68)	Yes
Ditmyer et al. 2010[19]	CS	4169	12 to 19	Postnatal	Permanent	Highest DMFT group	Yes
						OR = 1.42 (1.03–1.53)	
						Exposed DMFT = 7.23±0.06	
						Non-exposed DMFT = 6.74±0.10	
Hanioka et al., 2008[20]	CS	711	3	Postnatal	Primary	OR = 2.25 (1.51–3.37)	Yes
						Exposed dt = 2.1 (1.7–2.5)	
						Non-exposed dt = 1.2 (0.8–1.6)	
Iida et al., 2007[21]	CS	1576	2 to 5	Prenatal	Primary	OR: 1.68 (1.01–2.79)	Yes
Julihn et al., 2009[37]	L	15538	13 to 19	Postnatal	Permanent	Interproximal caries	Yes
						OR = 1.33 (1.22–1.44)	
Leroy et al., 2008[22]	CS	2533	3	Postnatal	Primary	a: OR = 1.98 (0.68–5.76)	Yes
			5	Postnatal	Primary	b: OR = 3.36 (1.49–7.58)	
Majorana et al., 2014[23]	CS	2395	2–3	Postnatal	Primary	Severe caries	Yes
						OR = 1.62 (1.34–1.96)	
Nakayama et al., 2015[24]	CS	1801	3	Postnatal	Primary	OR = 1.91 (1.43–2.54)	Yes
						Exposed dfs = 1.27±2.98	
						Non-exposed dfs = 0.53±1.69	
Nayani et al., 2018 [34]	CS	500	5–14	Postnatal	Permanent	Prevalence ratio <30min = 1.25 (1.07–1.45)	Yes
						Prevalence ratio >30min = 1.34 (1.05–1.35)	
Pita-Fernández et al., 2011[25]	CS	281	6 to 10	Postnatal	Primary	a: OR = 1.12 (0.55–2.28)	No
				Postnatal	Permanent	b: OR = 1.47 (0.62–3.47)	
Plonka et al., 2012[38]	L	1017	2–3	Postnatal	Primary	Predictive variable in a logistic model	Yes
Shenkin et al., 2004[39]	L	637	4 to 7	Postnatal	Primary	OR = 3.38 (1.68–6.79)	Yes
Shulman et al. 2005[26]	CS	7779	2 to 6	Postnatal	Primary	Exposed dft = 3.19 (1.03)	No
						Non-exposed dft = 2.10 (0.17)	
Tanaka et al., 2006[27]	CS	925	1 to 14	Postnatal	Primary	OR = 1.26 (0.93–1.69)	No
Tanaka et al., 2009[28]	CS	2015	3	Prenatal	Primary	a: OR = 1.43 (1.07–1.91)	Yes
				Postnatal		b: OR = 1.25 (1.04–1.50)	
Tanaka et al., 2010[29]	CS	20703	6 to 15	Postnatal	Permanent	OR = 1.29 (1.24–1.34)	Yes
Tanaka K et al., 2015[30]	CS	6412	3	Prenatal	Primary	a: OR = 1.70 (1.15–2.48)	Yes
				Postnatal	Primary	b: OR = 1.23 (1.05–1.45)	
Tanaka S et al., 2015[40]	RC	76920	3	Prenatal	Primary	a: HR = 1.46 (1.40–1.52)	Yes
				Postnatal	Primary	b: HR = 2.14 (1.99–2.29)	
Wiener et al., 2013[31]	CS	91642	1–15	Postnatal	Permanent	Special health care needs children	Yes
						OR = 1.23 (1.02–1.50)	
Williams et al., 2000[32]	CS	749	3 to 15	Postnatal	Primary	OR: 1.54 (1.07–2.21)	Yes

RC: retrospective cohorts; CS: cross-sectional; CC: case controls; L: longitudinal; OR: odds ratio; HR: hazard ratio; DMFT: permanent teeth decayed, missing and filled; DS: permanent tooth surfaces decayed; ds: primary tooth surfaces decayed; dft: primary teeth decayed and filled; dfs: primary tooth surfaces decayed and filled.

Most of their sample sizes were large, ranging from 180 in the study by Avçar et al.[35] to 76920 in Tanaka S et al.[40] and 91642 in Wiener et al.[31].

Only 5 articles investigated prenatal exposure (in the womb) [21,28,30,40,41] in the remaining 23 the exposure was postnatal (in infancy). Most of the studies examined caries in primary dentition[14,15,20–28,30,32,35,36,38–41], while the rest examined the permanent dentition. Different indices were used, such as DMFT or DS for permanent dentition and dft, ds or dt for primary dentition. Some odds ratios were calculated for the group with caries in second molars (Ayu-Yusuf et al.[17]), for those with the highest DMFT (Ditmyer et al.[19]) or for special health care needs children (Wiener et al.[31]); the rest of the studies did not effect any special selection.

On analysing the conclusions of each of the studies, it was observed that 80% of the articles included (in other words, all except five) concluded that an association does exist between passive smoking and a greater presence of caries in children, both in primary and in permanent dentition. Of the six studies that did not reach this conclusion, Aligne et al.[15] found an association with postnatal exposure for primary dentition but not for permanent dentition; Almerich-Torres et al.[16] and Bakhurji et al. [33] found no association between postnatal exposure and caries in permanent dentition in children 12–15 years old; Pita-Fernández et al.[25] found no association in either primary or permanent dentition, and Shulman et al.[26] and Tanaka et al.[27] did not find an association between postnatal exposure to environmental tobacco use and caries in primary dentition.

The quality assessment of the studies included in the systematic review is shown in Table 2. The quality of 43% of the articles included was medium and that of 57% was high. None of the studies presented low quality.

**Table 2:** Quality of the studies on the Newcastle-Ottawa scale.

*Study*	Selection *****/****	Comparability **	Exposure/Outcome ***	*Total Stars*
Aida et al., 2008[14]	****	**	**	8
Aligne et al., 2003[15]	*****	**	**	8
Almerich et al., 2013[16]	*****	*	**	8
Avçar et al., 2008[35]	****	**	***	9
Ayo-Yusuf et al., 2007[17]	****	**	**	8
Bakhurji et al., 2017 [33]	***	**	**	7
Bernabé et al., 2017[36]	*****	*	***	8
Carbajosa et al., 2011[18]	****	*	**	7
Claudia et al., 2016 [41]	***	**	***	8
Ditmyer et al. 2010[19]	*****	*	***	8
Hanioka et al., 2008[20]	***	*	***	7
Iida et al., 2007[21]	****	*	**	7
Julihn et al., 2009[37]	****	**	***	9
Leroy et al., 2008[22]	*****	**	**	9
Majorana et al., 2014[23]	****	*	**	7
Nakayama et al., 2015[24]	*****	*	***	9
Nayani et al., 2018 [34]	****	**	**	8
Pita-Fernández et al., 2011[25]	***	*	***	7
Plonka et al., 2012[38]	*****	*	***	9
Shenkin et al., 2004[39]	***	**	**	7
Shulman et al. 2005[26]	****	*	**	7
Tanaka et al., 2006[27]	****	*	*	7
Tanaka et al., 2009[28]	****	**	**	8
Tanaka et al., 2010[29]	****	**	***	9
Tanaka K et al., 2015[30]	****	*	**	7
Tanaka S et al., 2015[40]	****	*	**	7
Wiener et al., 2013[31]	****	*	**	7
Williams et al., 2000[32]	****	**	**	9

### Quantitative analysis

An overall meta-analysis was performed with data (odds ratios) from 21 studies (7 studies presented data from cohorts of different ages). Another three meta-analyses were performed with odds ratios data for the following subgroups: presence of caries in primary dentition in children exposed to tobacco use in the womb or prenatally (4 studies), presence of caries in primary dentition in children exposed to tobacco use postnatally (13 studies), and presence of caries in permanent dentition in children and adolescents exposed to tobacco use postnatally (1 studies, of which one presented data from two cohorts of different ages).

#### Association (OR) between the presence of caries in children and adolescents and exposure to tobacco use

**Fig 2:**
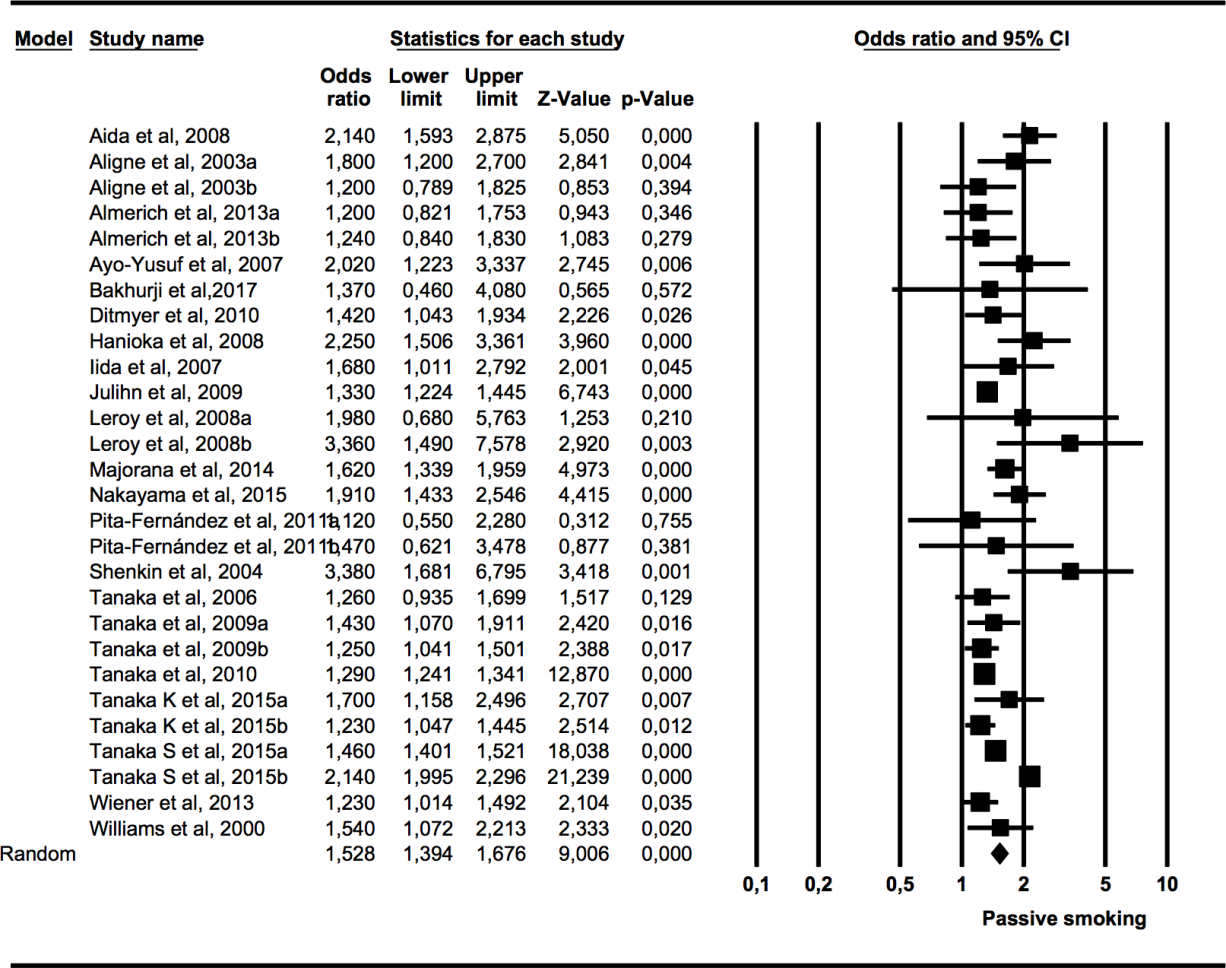
Forest plot of OR of caries presence in primary or permanent dentition of children and adolescents exposed to tobacco use pre- or postnatally.

Data were included from 21 studies using the OR to measure the association between caries in the primary and permanent dentition of children and adolescents and their pre-or postnatal exposure to tobacco use (Fig 2). The meta-analysis presented *Q* = 200.3 (p = 0.000) and I^2^ = 86.52%, indicating high heterogeneity. Using the random effects model, an OR of 1.53 with a 95% of CI 1.39–1.68 was calculated, which was statistically significant (Z test p-value = 0.000) and signifies that the presence of caries in children and adolescents exposed to tobacco use was 1.5 times higher than in the non-exposed children.

#### Association (OR) between caries presence in primary dentition and prenatal exposure to tobacco use in children

**Fig 3:**
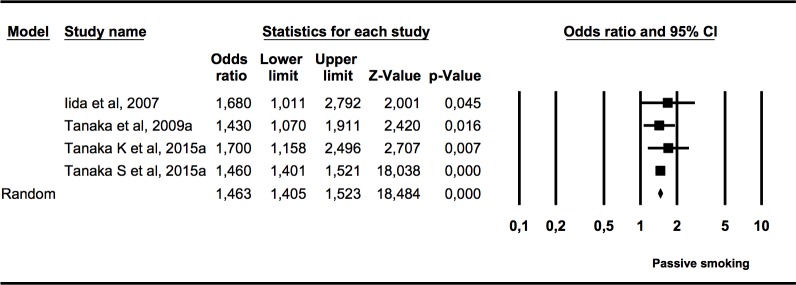
Forest plot of OR of caries presence in primary dentition of children exposed to tobacco use prenatally.

The first subgroup meta-analysis (Fig 3) included four studies that measured the presence of caries in primary dentition in children who had been exposed to tobacco use in the womb. Heterogeneity was not found (*Q* = 0.91, p = 0.824; I^2^ = 0%). The OR of 1.46 (95% CI 1.41–1.52) was statistically significant (Z test p = 0.000) and it was estimated that children exposed to tobacco use in the womb had 1.46 times more caries in primary teeth than the non-exposed group.

#### Association (OR) between caries presence in primary dentition and postnatal exposure to tobacco use in children

**Fig 4:**
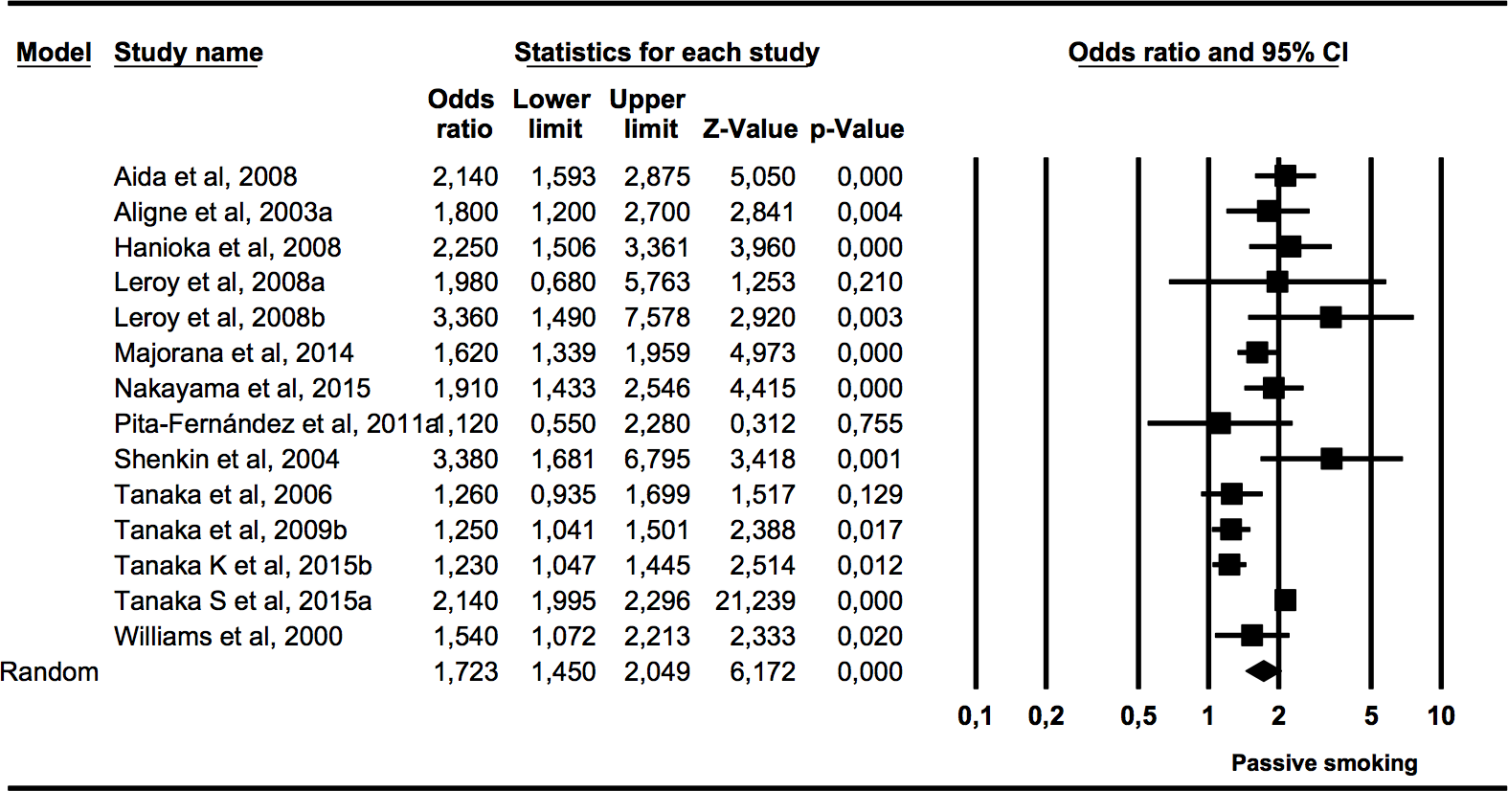
Forest plot of OR of caries presence in primary dentition of children exposed to tobacco use postnatally.

The second subgroup meta-analysis (Fig 4) included 13 studies that measured the presence of caries in primary dentition in children who had been exposed to tobacco use in infancy. The heterogeneity was high (*Q* = 76.59, p = 0.00; I^2^ = 83.01%). The estimate obtained through the combined random effects model was statistically significant (Z test p value = 0.000), with an OR of 1.72 (95% CI 1.45–2.05), meaning that the presence of caries in primary dentition in children exposed to tobacco use in infancy was 1.7 times higher than in the non-exposed children.

#### Association (OR) between caries presence in permanent dentition and postnatal exposure to tobacco use in children and adolescents

**Fig 5:**
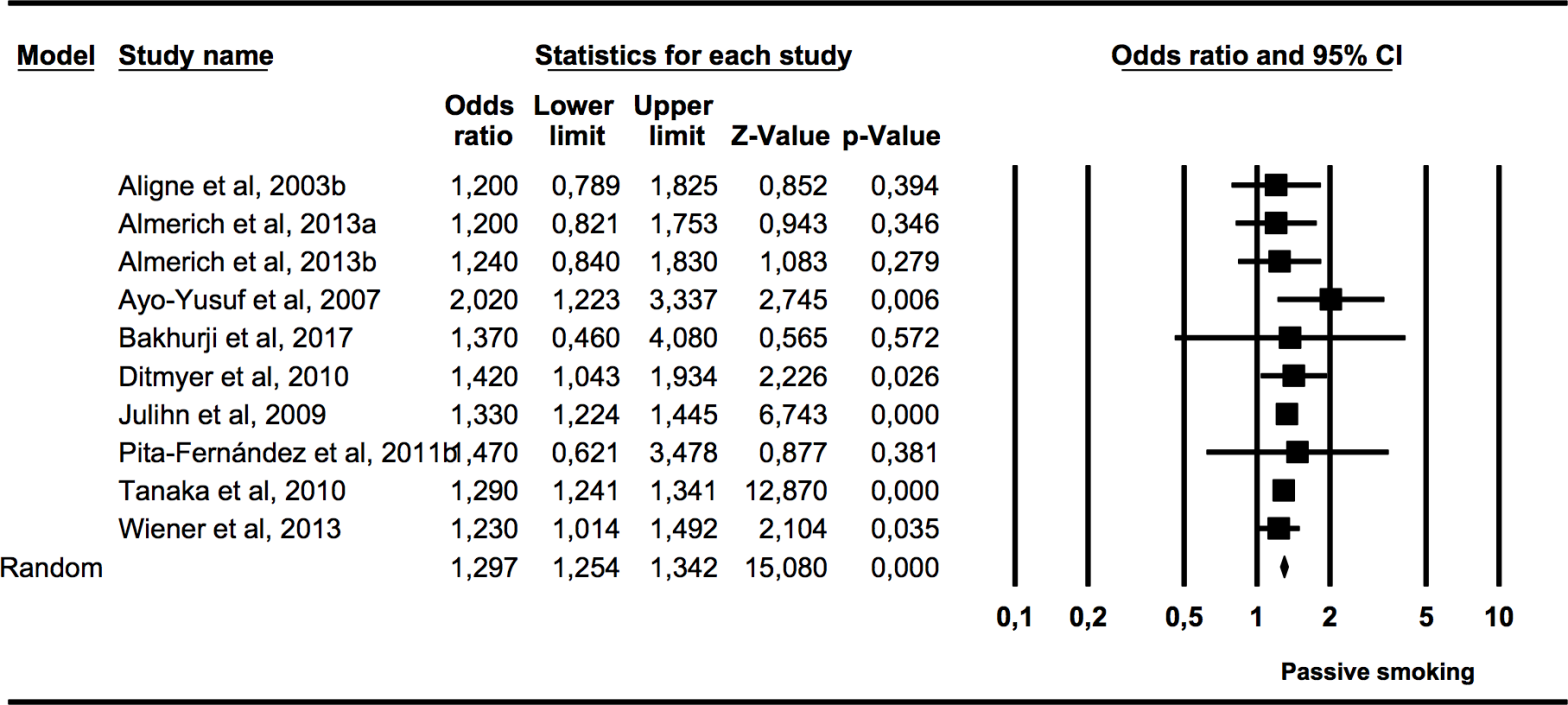
Forest plot of OR of caries presence in permanent dentition of children and adolescents exposed to tobacco use postnatally.

The third subgroup meta-analysis (Fig 5) included data from nine studies measuring the presence of caries in the permanent dentition of children and adolescents who had been exposed to tobacco use in infancy. Heterogeneity was not found (*Q* = 4.48; p = 0.88; I^2^ = 0%).

The calculation was statistically significant (p = 0.000), with an OR of 1.30 (95% CI 1.25–1.34), meaning that the presence of caries in children and adolescents exposed to tobacco use in infancy was 1.3 times higher than in the non-exposed children.

### Sensitivity analysis

**Fig 6:**
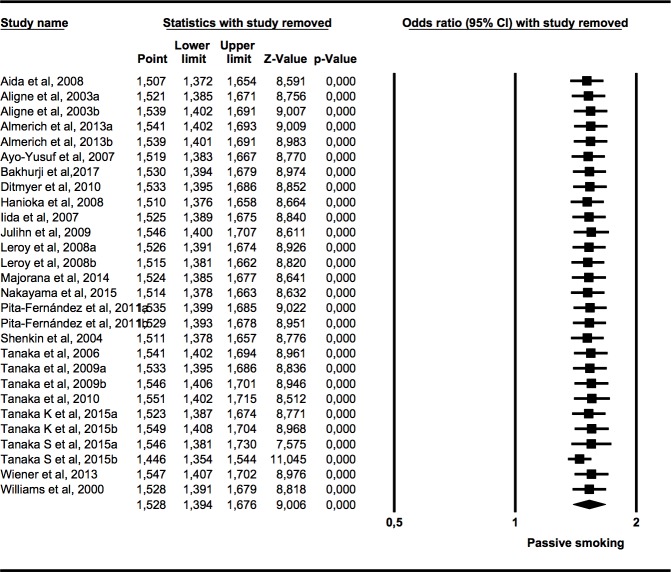
Forest plot “one-study removed” of OR of caries presence in primary or permanent dentition of children and adolescents exposed to tobacco use pre- or postnatally.

The one-study removed method did not detect any study that significantly affects the overall effect size estimated (Fig 6).

### Publication bias

The overall meta-analysis gave a *classic fail-safe number of 3434*, so a very high number of studies would be needed to invalidate the statistical significance of the present results. Egger’s regression intercept was 0.670 (p = 0.323). For the first subgroup meta-analysis, the *classic fail-safe number was 161* and Egger’s regression intercept was 0.453 (p = 0.278).

The classic fail-safe numbers for the second and third subgroup meta-analyses were 897 and 241, respectively. Their Egger’s regression intercept values were -0,893 (p = 0.394) and 0,233 (p = 0.442), respectively.

If we adjust the estimation of the meta-analysis using the Duval and Tweedie’s trim and fill, the estimated odds ratio for the overall meta-analysis is 1.50 (1.36–1.64). For the first subgroup, the estimated odds ratio is 1.46 (1.40–1.52), for the second subgroup is 1.72 (1.45–2.05) and 1.30 (1.25–1.33) for the third subgroup. No significant difference is observed between the adjusted odds ratio and the original estimations.

**Fig 7:**
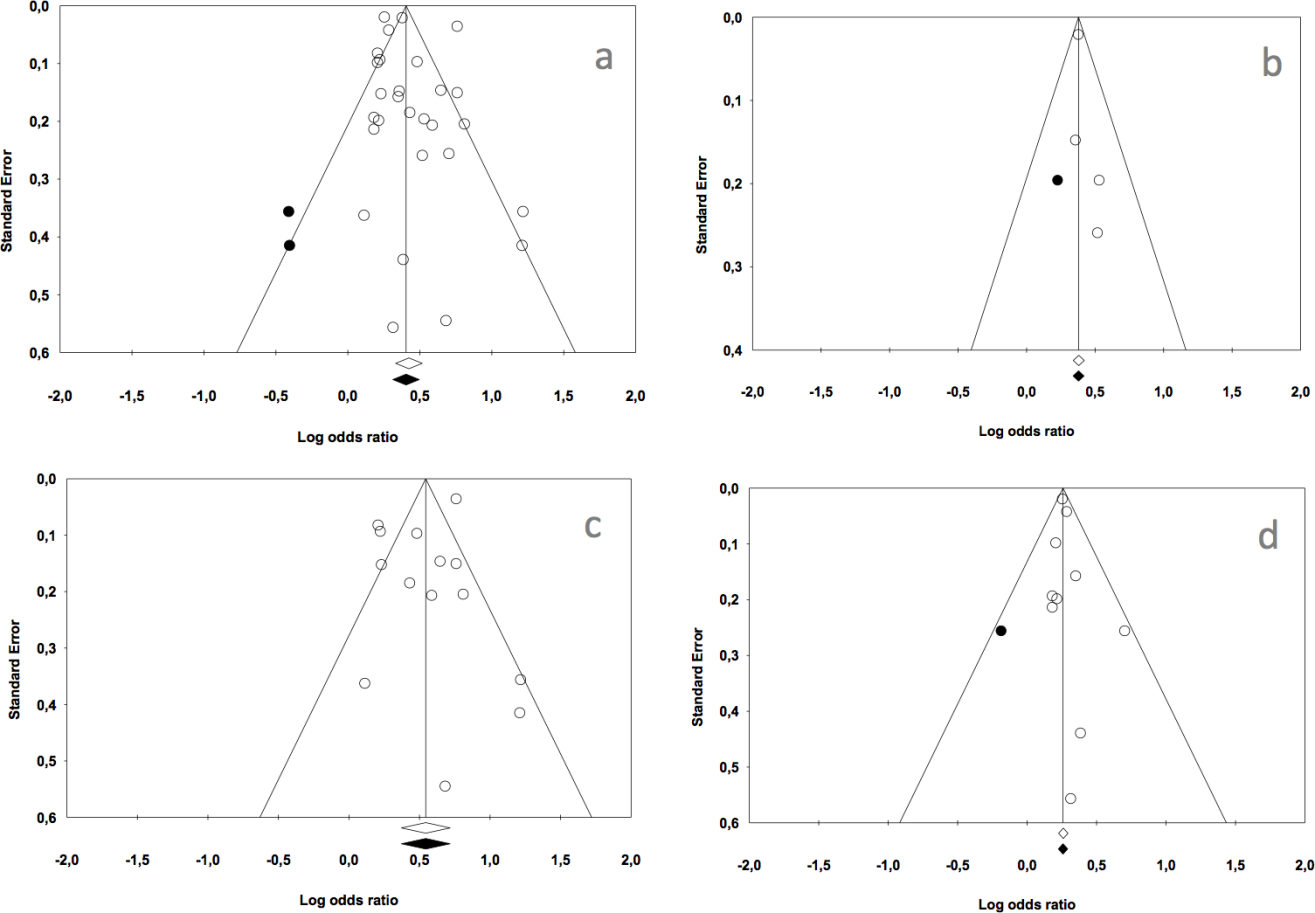
Funnel plots. a: Caries presence in primary or permanent dentition of children and adolescents exposed to tobacco use prenatally or postnatally. b: Caries presence in primary dentition of children exposed to tobacco use prenatally. c: Caries presence in primary dentition of children exposed to tobacco use postnatally. d: Caries presence in permanent dentition of children and adolescents exposed to tobacco use postnatal.

The funnel plots for the four meta-analyses are shown in Fig 7.

## Discussion

The present results confirm the existence of an association between passive tobacco exposure and dental caries in children and adolescents, for both prenatal and postnatal exposure and both primary and permanent dentition.

Most of the studies included in the meta-analysis were cross-sectional, making it impossible to study causal relation, although the four longitudinal studies[36–39] and the retrospective cohort study[40] concluded that this association did exist.

Several hypotheses support the biological plausibility of the association and could explain the causal mechanism of caries due to passive tobacco exposure. Chowdhury and Bromage[42] and Heikkinen et al.[43] state that exposure to tobacco use directly affects both the mineralisation of the developing tooth and the microorganisms, while Arora et al.[44] consider that exposure to environmental cadmium through cigarettes could play an important role. Impairment of the immune system could facilitate colonisation by Streptococcus mutans[45] and the lowering of vitamin C levels detected in the exposed children[46]. Avçar et al.[35] observed that children exposed to tobacco use had lower salivary pH, buffer capacity and saliva flow than non-exposed children, leading to a reduction in the capacity of saliva to protect against caries; this, together with a rise in *S mutans* and *Lactobacilli* levels, could explain the cause and effect relationship between tobacco and caries. In addition, according to Lindemeyer et al.[45], mothers who are smokers and have high levels of cariogenic bacteria transmit them more easily to their children in the first few years of life.

Despite all these plausible aetiological mechanisms, the tendency is to consider that the association between passive exposure to tobacco use and caries in children is mainly due to shared socio-economic, educational or behavioural factors. It is clear that children from a low socio-economic level show significantly higher caries rates[2].

Jakhete and Gitterman[47] found that exposure to tobacco use and poor nutrition were associated with higher caries prevalence in children from a low socio-economic level. Delpisheh[48] confirmed that passive exposure to tobacco use in children is significantly associated with low socio-economic level. Hanioka et al.[20] and Nakayama et al. [24] observed that the children of parents who smoked had poorer hygiene habits, brushed their teeth less frequently and consumed more sugar, favouring the appearance of caries. Leroy et al.[22] detected that children growing up with smoker parents brushed their teeth less and ate more between meals, so had poorer oral hygiene. Majorama et al.[23] found that children who were bottle-fed, lived in families from a low socio-economic level and were exposed to tobacco use had a greater likelihood of suffering severe caries.

The strong relationship between caries and socio-economic and behavioural factors does not help to clarify the relationship between passive exposure to tobacco use and caries in children. However, the estimate of this association in the present results is mostly derived from studies with OR values adjusted through logistic regression using variables such as toothbrushing frequency, age, gender, educational level, application of fluoride, visits to the dentist, socio-economic level, place of residence, etc. [14,15,17,19–23,25,26,29,39]. Few studies that presented only the raw OR were included in the meta-analysis [16,35]. Consequently, this confirms not only the strength of the association but also its independence from the other factors that have been related to the aetiology of dental caries.

The dose-response relationship between levels of exposure to tobacco use and dental caries has been studied by several authors [14,15,20,28,29,34], all of whom have confirmed this relationship. The studies by Tanaka et al.[28,29] examined exposure in terms of years of smoking in the household, number of cigarettes smoked and whether smoking had taken place only during the child’s infancy or also during the pregnancy. With the exception of Tanaka et al.[29], who focused on permanent dentition, all the studies examined the presence of caries in primary dentition. Nayani et al. [34]observed a higher prevalence ratio in children exposed more than 30 minutes to tobacco smoke than in children exposed less time. Aligne et al.[15] established a significant positive relationship between blood cotinine levels and the odds ratio for caries presence.

An important point to note is how exposure is measured. Most of the studies were based on questionnaires addressed to the parents, which could affect the results by introducing memory or response biases. Cotinine, on the other hand, is a primary metabolite of nicotine with a longer half-life and is considered a reliable biomarker when screening for passive exposure to tobacco use. It can be measured in body fluids such as plasma, saliva and urine[49]. For this reason, measuring the cotinine level is recommended in addition to collecting information from the parents’ answers to the questionnaires. Despite this, only two of the studies included (Aligne et al.[15] and Avçar et al.[35]) used cotinine levels to quantify passive exposure to tobacco use.

When analysing the data in the present study, three subgroup meta-analyses were performed to check for heterogeneity, distinguishing between exposure in the womb (prenatal) or in infancy (postnatal) and between caries in primary teeth and in permanent teeth. No studies that examined prenatal exposure and caries in permanent teeth were identified. The only meta-analysis that showed heterogeneity was that for association between postnatal exposure and caries in primary teeth, even though the large sample size of most of the studies led to narrow OR confidence intervals, which facilitate heterogeneity between the studies. The classic fail-safe numbers and Egger regression values indicated little risk of publication bias in the present results.

The cross-sectional design of most of the studies did not allow confirmation of a possible aetiology of passive exposure to tobacco use, despite the association found with caries, the positive dose-response relationship, the possible hypotheses of biological plausibility, and even that the few longitudinal studies included also showed this association. Prospective studies are needed to identify passive tobacco exposure in the home, measured through the joint use of questionnaires and cotinine level testing, as a direct risk factor for caries in children.

## Conclusions

In view of the foregoing, after examining the available evidence and bearing in mind the above-mentioned methodological limitations, it may be asserted that a moderate association (estimated OR: 1.5) between the presence of caries in primary and permanent dentition and passive exposure (prenatal or postnatal) to tobacco smoke in children and adolescents does exist.

## Supporting information

S1 Checklist PRISMA 2009 checklist. (DOC)
